# Monocyte-macrophage membrane expression of IL-1R2 is a severity biomarker in sepsis

**DOI:** 10.1038/s41419-025-07597-x

**Published:** 2025-04-10

**Authors:** Domenico Supino, Sadaf Davoudian, Rita Silva-Gomes, Daniele Piovani, Roberto Garuti, Antonio Desai, Sarah N. Mapelli, Francesco Scavello, Silvia Carnevale, Andrea Mariancini, Elena Magrini, Roberto Leone, Marina Sironi, Sonia Valentino, Diletta Di Mitri, Federica Portale, Carlo Fedeli, Denise Comina, Stefanos Bonovas, Antonio Voza, Alberto Mantovani, Barbara Bottazzi, Cecilia Garlanda

**Affiliations:** 1 IRCCS Humanitas Research Hospital, Milan, Italy; 2 Life and Health Sciences Research Institute (ICVS), School of Medicine, University of Minho, Braga, Portugal; 3 ICVS/3B’s-PT Government Associate Laboratory, Guimarães/Braga, Portugal; 4 Department of Biomedical Sciences, Humanitas University, Milan, Italy; 5 Department of Emergency, IRCCS Humanitas Research Hospital, Milan, Italy; 6 The William Harvey Research Institute, Queen Mary University of London, London, UK

**Keywords:** Infectious diseases, Biomarkers

## Abstract

Interleukin-1 (IL-1)/IL-1 receptor family consists of activators and inhibitors which play a key role in inflammation, emergency myelopoiesis, and myeloid cell activation. The latter includes the IL-1R2 decoy receptor. To investigate the expression and significance of IL-1R2 in sepsis, we conducted high-dimensional flow cytometry of circulating cells from patients stratified according to the Sequential Sepsis-Related Organ Failure Assessment (SOFA) score. Here we report that the IL-1 decoy receptor is selectively upregulated on the plasma membrane of leukocytes and, in particular, monocytes from septic patients, and downregulated in septic shock. Flow cytometry combined with transcriptomic analysis of publicly available datasets indicated that IL-1R2 is associated with the differentiation of monocytes to a population of circulating monocytic cells with macrophage features (Mono/M*φ*). In vitro stimulation of monocytes from healthy donors with Colony Stimulating Factors (CSFs), in particular GM-CSF and Lipopolysaccharides (LPS), induced IL-1R2^+^ Mono/M*φ*, which recapitulated the characteristics of sepsis-associated monocytic cells, including low expression of HLA-DR, high levels of macrophage markers such as MS4A4A and CD63, immune checkpoints, immunosuppressive molecules and selected scavenger receptors. Membrane-associated IL-1R2 and MS4A4A correlated with immunological markers, cytokine storm, and clinical parameters (e.g., SOFA score, creatinine, survival), reflecting the infection severity in hospitalized patients.

Thus, in sepsis IL-1R2 is expressed in a subset of circulating monocytes co-expressing mature macrophage and immune dysfunction features with clinical significance.

## Introduction

Sepsis is a complex life-threatening syndrome, determined by dysregulated host response to pathogens, which leads to multiple organ dysfunction, shock, and death [1–4]. This multifactorial condition still represents an important cause of death worldwide, particularly in the elderly population [2–6].

In the context of infection, the IL-1 system plays a key role in the activation of inflammation and, in turn, of anti-microbial responses [7]. The IL-1 system consists of eight agonist ligands, three receptor antagonists, and a large receptor family (ILRs) including the negative regulators IL-1R2 and IL-1R8 [7]. IL-1R2 lacks a signaling domain and acts as a decoy receptor for IL-1, both as a cell-associated receptor and as a soluble form (sIL-1R2), by interacting with the ligand and with IL-1R3, the accessory protein of the signaling IL-1R1 [8]. In septic conditions, negative regulation of IL-1 activity by the IL-1 receptor antagonist (IL-1Ra) has therapeutic efficacy in patients with macrophage activation syndrome and in patients with COVID-19 [9–11], whereas for IL-1R2, available information suggests a role as biomarker of severity or immune suppression. sIL-1R2 has been recently shown to correlate with the SOFA score and mortality [12], and the transcript for IL-1R2 has been associated with sepsis-associated signatures in both monocytes and neutrophils [13, 14]. In line with the dual role of acute inflammation in microbial control and immunopathology, contrasting results have been reported regarding the function of IL-1R2. In particular, upregulation and shedding of IL-1R2 were associated with impaired antimicrobial activity of monocytes, contributing to infection severity [15, 16].

In the effort to dissect the expression and regulation of IL-1R2 in sepsis, we performed a high-dimensional flow cytometric analysis of peripheral blood mononuclear cells (PBMCs) from non-sepsis, sepsis, and septic shock patients and combined it with transcriptomic analysis, as well as in vitro assays. Here we show that IL-1R2 was upregulated particularly in monocytic cells from septic patients, correlating with severity, survival, and parameters of immune dysfunction. IL-1R2-expressing monocytic cells displayed a distinctive transitory signature from monocytes to macrophages associated with immune suppression markers, which was reproducible by stimulating circulating monocytes with CSFs and LPS. These results suggest that IL-1R2 expression by monocytes may be exploited as a biomarker of severity and immune dysregulation in septic conditions.

## Materials and methods

### Study population

A prospective observational study was conducted by enrolling 178 adult patients (18 years old or more) admitted to the Emergency Department (ED) of Humanitas Research Hospital between October 2017 and February 2020 for suspected sepsis [12]. Identification of patients was based on the Sepsis-3 criteria defined by the Third International Consensus Definitions for Sepsis and Septic Shock [1]. PBMCs were available for a total of 117 patients with suspected sepsis. According to Sepsis-3 criteria, enrolled patients with a SOFA ≥ 2 were considered septic individuals (*n* = 94) and were further subdivided in sepsis (*n* = 69) and septic shock (*n* = 25) patients. Patients with SOFA score < 2 diagnosed with infectious disease were assigned to the “non-sepsis” group (*n* = 23). The cohort included subjects aged 35–98 years old and composed of 64% men and 36% women. Demographic information (age, sex, and prior medical history), ED stay, hospital stay, Intensive Care Unit (ICU) admission, and blood pressure were collected and documented. A group of healthy controls (HC) (*n* = 19) was enrolled among volunteers with median age and 25% and 75% quartiles (Q1–Q3) 58 years (54–64).

### Sample collection and preparation

Blood samples of patients with suspected sepsis were collected in EDTA tubes at arrival in the ED of Humanitas Research Hospital. Blood tubes were centrifuged at 1800 rpm for 10 min to remove plasma, then PBMCs were isolated by Ficoll density gradient centrifugation. Purified PBMCs were conserved in Fetal Bovine Serum (FBS) with 10% DMSO at −80 °C until further analysis.

### Flow cytometry

PBMCs were stained for flow cytometry analysis. Extracellular staining was performed using PBS containing 2% FBS, 2 mM EDTA, and 0.05% NaN_3_. Cell viability was determined by Aqua LIVE/Dead-405 nm staining (Invitrogen) or Fixable Viability Dye (FVD) eFluor 780 (eBioscience). To block non-specific receptor binding, cells were incubated for 10 min at 4 °C with human FcR blocking reagent (Miltenyi Biotec). Finally, samples were incubated with the antibody mix for 20 min at 4 °C. Anti-CD121b, Clone REA744 (Miltenyi Biotec), was used to stain IL-1R2. The complete list of antibodies used is reported in Table S1. The gating strategy is shown in Fig. S1. For intracellular staining, cells were permeabilized using the Foxp3/Transcription Factor Staining Buffer Set (ThermoFisher) for 30 min at room temperature, followed by 1 h incubation with the antibody mix. The investigator who conducted the flow cytometry analysis was blinded to the group allocation. An aliquot of PBMCs from the same healthy donor was used as an internal control in flow cytometry analysis of the infected patients. Results are reported as frequency or as median fluorescence intensity (MFI).

Cells were analyzed on LSR Fortessa (BD Bioscience) or FACSymphony (BD Bioscience). Data were analyzed with FlowJo software (Treestar, Ashland, US).

### Monocyte isolation and stimulation

Monocytes were isolated from buffy coats from healthy donors using the CD14 MicroBead Kit (Miltenyi Biotec). The cells were primed for 4 days with 50 ng/mL of recombinant human (rh) Macrophage Colony-Stimulating Factor (M-CSF) or rh Granulocyte-Macrophage Colony-Stimulating Factor (GM-CSF) (Peprotech) in RPMI 1640 (Lonza) with 10% FBS (Lonza), 1% l-glutamine (Lonza) and 1% Penicillin-Streptomycin (Lonza). On day 4, cells were stimulated with 100 ng/mL of 055:B5 LPS (Sigma), in the presence of GM-CSF or M-CSF for 24 h. BD GolgiPlug™ (containing Brefeldin) was further added 4 hours prior to intracellular staining.

### Quantitative PCR

RNA was purified using Direct-zol RNA Microprep kit (Zymo Research). cDNA was synthesized by reverse transcription using High High-Capacity cDNA reverse transcriptase kit (Applied Biosystems). Quantitative real-time PCR was performed using the SYBR Green PCR Master Mix (Applied Biosystems) in a QuantStudio 7 Flex Real-Time PCR System (Thermo Fisher Scientific). Data were evaluated with the 2^−ΔCT^ method, conducting the normalization based on *GAPDH*. Primers were designed according to the published sequences and listed as follows: *CD14* Forward 5′-GACCTAAAGATAACCGGCACC-3′; *CD14* Reverse 5′-GCAATGCTCAGTACCTTGAGG-3′; *IL1R2* Forward 5′-CCGCATCAACCTGACATGG-3′; *IL1R2* Reverse 5′- GCCCACATCCGTGTCTCTT-3′; *MS4A4A* Forward 5′- CTGGGAAACATGGCTGTCATA-3′; *MS4A4A* Reverse 5′- CTCATCAGGGCAGTCAGAATC-3’; *GAPDH* Forward 5′-GGAGCGAGATCCCTCCAAAAT-3′; *GAPDH* Reverse 5′-GGCTGTTGTCATACTTCTCATGG-3′.

### Cytokines and soluble mediators

Circulating levels of sIL-1R2, PTX3, IL-10, IL-6, IL-1β, TNF-α, IL-18, IL-8, and IL-1ra were previously analyzed as described [12] by ELISA or ELLA Automated Immunoassay System (ProteinSimple), and results were used in this study for correlation analysis with flow cytometry markers.

### Bioinformatic analysis

The analysis was performed using data from a single-cell RNA sequencing (scRNA-seq) study of PBMCs of septic patients with urinary tract infections [13]. In this study, IL-1R2 was overexpressed in a specific cluster of IL-1R2^+^ HLA-DR^low^ monocytes (named “MS1”). Differentially Expressed Genes (DEGs) were extrapolated by comparing the MS1 cluster to the IL-1R2^low^ HLA-DR^+^ MS2 cluster (MS1 vs MS2 dataset) or MS1 cluster to all the other monocytes (MS1 vs All Monocyte dataset). State markers were based on false discovery rate (FDR) [13]. Normalized transcript expression values were extrapolated from the Human Protein Atlas dataset, Single Cell Type Database, Blood & Immune cells (updated on 13/10/2022) (https://www.proteinatlas.org/humanproteome/single+cell+type).

Relative expression in monocytes versus macrophages was calculated using the formula: Normalized transcript expression values (nTPM) of Macrophages—nTPMs of Monocytes/nTPMs of Macrophages + nTPMs of Monocytes.

Epigenetic profiling of *IL1R2* was conducted by using publicly available Chromatin Immunoprecipitation (ChIP) sequencing data from ChIP-Atlas (https://chip-atlas.org/peak_browser). Cell types “Monocytes” and “Macrophages” were selected, and a Threshold for Significance of 200 was used. Data were represented by using Integrative Genomics Viewer (IGV).

### Statistical analysis

Quantitative data are presented as mean ± standard error of the mean (SEM) or percentage, when indicated. The D′Agostino–Pearson test was used to determine if the variables of interest were normally distributed. One-way ANOVA or Kruskal–Wallis test were used to compare multiple groups according to Gaussian distribution. Two-tailed multiple Student’s or Two-tailed Mann–Whitney *U*-test were used to compare unmatched groups according to Gaussian distribution. Correlations were assessed by Spearman’s rank correlation coefficient and the respective *p* value. Non-linear associations were assessed by employing fractional polynomial regression (STATA command: fracpoly). Sample size was then considered sufficient when differences of 20% or greater between the groups were detected (10% significance level and 80% power). Outliers were identified by ROUT methods (Q = 1%). The best-fitting second-degree fractional polynomial model was chosen by minimizing deviance in polynomial regression. The data underwent log10-transformation and were winsorized at the 1st and 99th percentiles before applying regression techniques to potentially optimize model fit and mitigate the weight of extreme outliers. For interpreting the results, the marginal effect was displayed. To estimate the sensitivity and specificity of IL-1R2, MS4A4A, and HLA-DR expression as markers in non-sepsis, sepsis, and shock patient cohorts, receiver operating characteristic (ROC) curve analysis was performed, and the Area Under the Curve (AUC) was calculated.

Association of IL-1R2 with survival in non-septic plus sepsis and in septic shock cohorts was assessed by Cox proportional hazards models after adjusting for sIL-1R2, IL-1β, IL-18, IL-1ra, and PTX3 plasma levels. Proportional hazards additive models with three degrees of freedom [17] were further applied to plot the partial effect of IL-1R2 expression on mortality.

Stata 16.1 (Stata Corp., College Station, TX, USA), R software, and GraphPad Prism 7.0 (GraphPad Software Inc., CA) were used for statistical analyzes and graphics. *P* values < 0.05 were considered statistically significant. All tests were two-sided. P values are reported in figures and tables as follows: ^*^p ≤ 0.05; ^**^p ≤ 0.01; ^***^p ≤ 0.001; ^****^p ≤ 0.0001.

## Results

### Characteristics of the population

Patients analyzed in the present study were part of a population of 178 subjects admitted to Humanitas Research Hospital [12]. In this cohort, a total of 117 patients were analyzed based on the availability of PBMCs. Patients were subdivided into non-sepsis (*n* = 23), sepsis (*n* = 69), and septic shock (*n* = 25) in accordance with the Sepsis-3 Consensus Definition of Sepsis [1]. 19 age-matched healthy controls were enrolled. Table S2 summarizes the demographic and clinical characteristics of patient cohorts with the exception of two non-sepsis individuals whose clinical data were not available. The median (Q1-Q3) age was 76 (68–84) years, and the percentage of male subjects (64%) was higher compared to females (36%). Median age of healthy controls was 58 years (54–64), 7 were males (37%), and 12 were females (63%).

Most of the sepsis patients were diagnosed with a respiratory origin infection (*n* = 31, 45%), while the urinary tract (*n* = 11, 44%) was the most common infection site in septic shock patients. Creatinine, urea, potassium levels, SOFA, and qSOFA scores increased from non-sepsis to sepsis and septic shock patients.

### Immunophenotyping of PBMCs in sepsis

PBMCs were analyzed by multiparametric flow cytometry analysis. The frequency of CD3^+^ T lymphocytes was very heterogeneous in patients and lower in non-sepsis and sepsis patients compared to healthy individuals, in agreement with relative lymphopenia in sepsis [4] (Fig. S2a). The reduction of T lymphocytes did not reach statistical significance in septic shock patients compared to healthy donors (Fig. S2a). The frequency of both conventional CD4^+^ T cells (Tconv) and regulatory T cells (Treg) was reduced in all patient groups (Fig. S2b, c). Conversely, CD8^+^ T cells, B lymphocytes, and NK cells (Fig. S2d–f) were not significantly affected. The CD4/CD8 ratio, a prognostic risk factor in infections, was reduced in all infected patients compared to healthy controls, but it was not associated with disease severity (Fig. S2g).

**Fig. 1:**
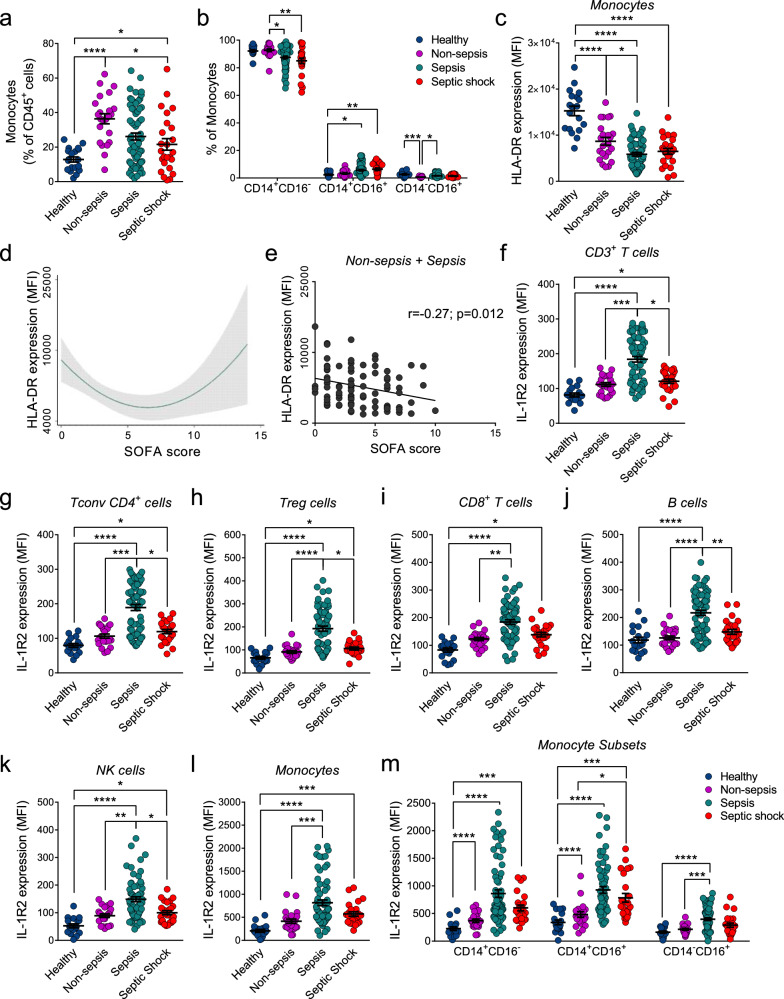
IL-1R2 expression and characterization of leukocytes in septic conditions. **a**–**d** Frequency of circulating monocytes (**a**), CD14^+^CD16^-^, CD14^+^CD16^+^ and CD14^-^CD16^+^ monocyte subsets (**b**), and HLA-DR protein expression (**c**) in PBMCs from the entire cohort. **d** Association between disease severity (SOFA score) and HLA-DR expression on monocytes in the entire cohort. The gray area represents the 95% confidence interval. **e** Correlation of monocyte HLA-DR expression with SOFA score in non-sepsis plus sepsis subgroups. Flow cytometry analysis of IL-1R2 expression in CD3^+^ T cells (**f**), Tconv CD4^+^ (**g**), Treg (**h**), CD8^+^ T cells (**i**), B cells (**j**), NK cells (**k**), total monocytes (**l**), and CD14^+^CD16^−^, CD14^+^CD16^+^ and CD14^−^CD16^+^ monocyte subsets (**m**). **a**–**m** Bars represent mean ± SEM. Kruskal–Wallis with Dunn’s multiple comparison test (**a**–**b**, **f**–**m**) or one-way ANOVA with Tukey multiple comparison test (**c**). ^*^p < 0.05, ^**^p < 0.01, ^***^p < 0.001, ^****^p < 0.0001. Healthy donors: *n* = 19; Non-sepsis patients: *n* = 23; Sepsis patients: *n* = 69; Septic Shock patients: *n* = 25.

As expected [5], the frequency of circulating monocytes, although very heterogeneous in patients, was higher in infected individuals (Fig. 1a), contributing to a lower CD3^+^/Monocytes ratio (Fig. S2h). Monocytes were further dissected into CD14^+^CD16^−^ classical monocytes, CD14^+^CD16^+^ intermediate monocytes, and CD14^-^CD16^+^ non-classical monocytes [18]. Increased frequency of CD14^+^CD16^+^ monocytes and reduced frequency of CD14^+^CD16^−^ monocytes was observed in septic and septic shock patients compared to healthy controls (Fig. 1b). In line with other studies [5, 13, 19, 20] HLA-DR was downregulated in monocytes of severely infected individuals compared to healthy donors, and was further reduced in sepsis and septic shock patients, negatively associating with the disease severity (Fig. 1c). In agreement, HLA-DR expression in monocytes was negatively correlated with the SOFA score, especially in non-sepsis and sepsis cohorts (Spearman *r* = −0.27, *p* = 0.012) (Fig. 1d, e and Table 1). When all the infected patients were considered, this correlation was lost (Spearman *r* = −0.16, *p* = 0.11, Table 1), due to the high heterogeneity observed in patients with SOFA score > 10.

**Table 1:** Correlation of IL-1R2, MS4A4A, and HLA-DR with clinical parameters.

Variables	Groups	HLA-DR		SOFA score		Creatinine (mg/dL)		Urea (mg/dL)	
		Spearman *r*	*p* value^*^	Spearman *r*	*p* value^*^	Spearman *r*	*p* value^*^	Spearman *r*	*p* value^*^
IL-1R2	Non-sepsis + Sepsis	−0.28	**0.0098**	0.47	**<0.0001**	0.41	**<0.0001**	0.22	**0.037**
	All infected patients	−0.3	**0.0015**	0.3	**0.0012**	0.35	**0.0002**	0.17	0.079
	All groups	−0.48	**<0.0001**						
MS4A4A	Non-sepsis + Sepsis	0.005	0.96	0.3	**0.0042**	0.19	0.071	0.075	0.49
	All infected patients	−0.04	0.69	0.025	0.84	0.21	**0.034**	0.11	0.26
	All groups	−0.26	**0.0043**						
HLA-DR	Non-sepsis + Sepsis			−0.27	**0.012**	−0.13	0.24	−0.13	0.23
	All infected patients			−0.16	0.11	−0.066	0.5	−0.12	0.2
	All groups								

^*^Spearman’s rank correlation.

In bold, *p* values < 0.05.

These data indicate that sepsis was associated with lymphopenia and monocytosis and that our cohort of patients met sepsis-associated immunopathological criteria. Downregulation of HLA-DR and increased frequency of CD14^+^CD16^+^ subset indicated that monocytes were not only quantitatively but also qualitatively affected by the disease severity.

### Expression of IL-1R2 in PBMCs in sepsis

We previously showed that plasma concentration of sIL-1R2 increases in patients with sepsis and septic shock, correlating with severity and mortality [12]. To address whether the membrane-IL-1R2 was also modulated in sepsis, its expression was evaluated by flow cytometry.

IL-1R2 was mostly expressed by monocytes, whereas lymphoid cells showed low levels of the protein in healthy conditions, which was upregulated reaching the highest levels in septic patients (Fig. 1f–m). The induction of IL-1R2 observed in CD3^+^ T cells from septic patients (2.3-fold increase compared to healthy donors) (Fig. 1f), was observed in all T cell subsets, including CD4^+^ (Fig. 1g, h) and CD8^+^ T cells (Fig. 1i). Increased expression of IL-1R2 was observed in B cells (1.8-fold increase) and NK cells (2.8-fold increase) from septic patients (Fig. 1j, k).

In monocytes, IL-1R2 was mostly upregulated in the sepsis cohort (3.96-fold increase) (Fig. 1l).

CD14^+^CD16^−^ and CD14^+^CD16^+^ monocyte subsets expressed the highest levels of IL-1R2 in healthy donors compared to non-classical monocytes CD14^-^CD16^+^ (Fig. 1m). In sepsis patients CD14^+^CD16^−^, CD14^+^CD16^+^, and CD14^−^CD16^+^ cells showed, respectively, 3.8-, 2.7-, and 2.5-fold increase of IL-1R2 MFI, compared to healthy subjects (Fig. 1m).

In both monocytes and lymphocytes, IL-1R2 cell surface expression was reduced in septic shock, compared to sepsis (Fig. 1f–m). Thus, further statistical analyzes were also performed, excluding septic shock patients.

Collectively, multiparametric flow cytometry analysis revealed upregulation of IL-1R2 on the plasma membrane, with the highest levels in sepsis.

### IL-1R2 is transcriptionally regulated during monocyte-to-macrophage differentiation

**Fig. 2:**
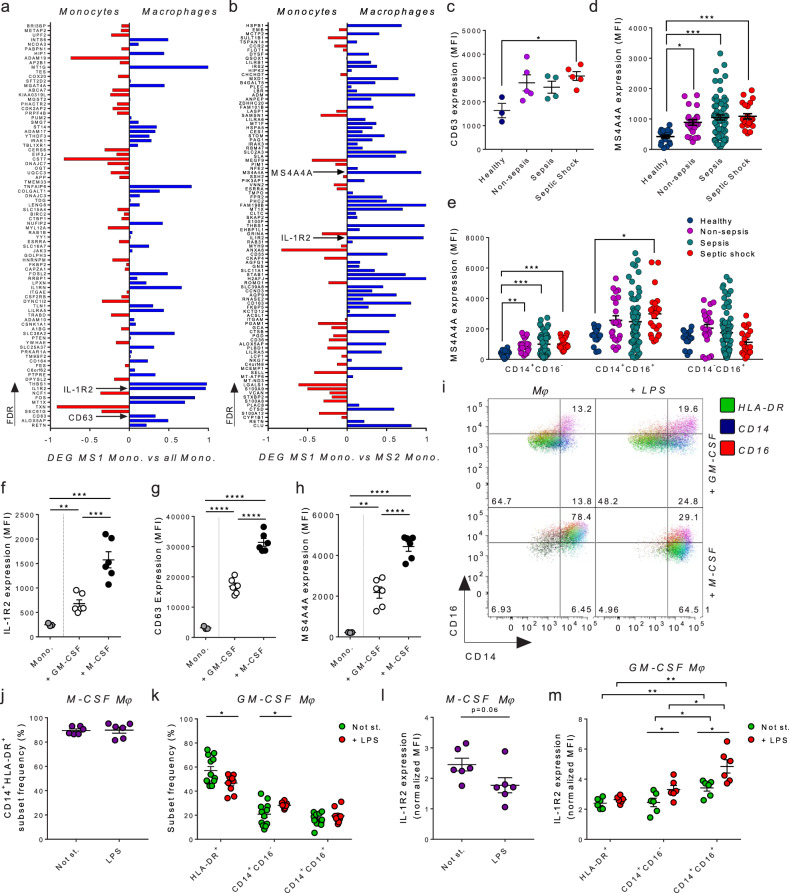
Association of IL-1R2 to Mono/M*φ* functional signatures in monocytic cells in sepsis. **a**, **b** Diagrams showing the expression in monocytes or macrophages of urosepsis monocytic cells (MS1) signatures. DEGs of MS1 Monocytes versus All Monocytes (**a**) or versus MS2 Monocytes (**b**) are shown. **c** CD63 protein expression in monocytes. MS4A4A protein expression in monocytes (**d**) and in CD14^+^CD16^−^, CD14^+^CD16^+^, and CD14^−^CD16^+^ monocyte subsets in the entire cohort (**e**). Flow cytometry analysis of IL-1R2 (**f**), CD63 (**g**), and MS4A4A (**h**) expression in unstimulated monocytes, GM-CSF- and M-CSF-induced Mono/M*φ*. **i** Representative polychromatic plot showing HLA-DR, CD14, and CD16 expression in GM-CSF- and M-CSF-induced monocytic cells. **j** Frequency of HLA-DR^+^ CD14^+^ subset in M-CSF-induced Mono/M*φ* after stimulation with LPS. **k** Frequency of HLA-DR^+^, CD14^+^CD16^−^ and CD14^+^CD16^+^ subsets in GM-CSF-generated Mono/M*φ* after stimulation with LPS. Expression of IL-1R2 in polarized M-CSF- (**l**) and GM-CSF-induced (**m**) Mono/M*φ* normalized on the respective unstimulated monocytes. **c**–**m** Bars represent mean ± SEM. One-way ANOVA with Tukey multiple comparison test (**c**); Kruskal–Wallis with Dunn’s multiple comparison (**d**, **e**); Two-tailed Student’s *t*-test (**F**–**H**, **J**–**M**); ^*^p < 0.05, ^**^p < 0.01, ^***^p < 0.001, ^****^p < 0.0001. **c** *n* = 3–5 per group. **d**, **e** Healthy donors: *n* = 19; Non-sepsis patients: *n* = 23; Sepsis patients: *n* = 69; Septic Shock patients: *n* = 25. **f**–**h** One representative experiment out of two with similar results is shown. *n* = 3 healthy donors, with technical duplicates of Mono/M*φ*. **m** *n* = 6, pooled data from two experiments are shown.

To better investigate IL-1R2 regulation, we focused on monocytes, taking advantage of a single-cell RNA sequencing (scRNA-seq) study of septic patients with urinary tract infections [13]. IL-1R2 was shown to be overexpressed by a cluster of IL-1R2^+^ HLA-DR^low^ monocytes (named MS1) emerging in the bloodstream as a result of sepsis-induced dysregulated myelopoiesis [13]. In particular, Differentially Expressed Genes (DEGs) were identified by comparing IL-1R2^+^ HLA-DR^low^ MS1 cluster vs All Monocytes (Fig. 2a) and vs the IL-1R2^low^ HLA-DR^+^ MS2 cluster (Fig. 2b). Having observed genes typically associated with macrophages (e.g., MS4A4A, CD63, CD163) [21–24] in the MS1 cluster, we further investigated potential differentiation of monocytes to macrophages in sepsis. We evaluated the expression of MS1 DEGs in monocytes and in macrophages using a publicly available dataset from the Human Protein Atlas. The expression was extrapolated as Normalized transcript expression values, or nTPM, and compared using the formula nTPM of Macrophages—nTPMs of Monocytes/nTPMs of Macrophages + nTPMs of Monocytes, which allowed us to represent transcripts with very heterogeneous expression on a relative scale from −1 to 1 (Fig. 2a, b).

Intriguingly, IL-1R2 was mainly expressed by macrophages (Fig. 2a, b), suggesting that it is associated with myeloid cell differentiation. Moreover, most of MS1 signature genes (> 55.68% in Fig. 2a, and >63.6% in Fig. 2b) were highly expressed by macrophages. Among the DEGs (Fig. 2a, b), we focused on MS4A4A, a tetraspan molecule associated with anti-inflammatory markers and involved in Dectin-1-dependent activation of macrophages [21]. The molecule is poorly expressed by monocytes and upregulated following differentiation into macrophages [22]. We also found CD63, a tetraspanin family member expressed by monocyte-derived macrophages [23] and negatively associated with survival of sepsis patients [25, 26]. In agreement with transcriptional data [13, 27], flow cytometry analysis revealed a significant upregulation of both CD63 and MS4A4A in monocytes from our cohort of patients (Fig. 2c, d). Specifically, MS4A4A expression was upregulated in monocytes of sepsis patients (Fig. 2e), and increased levels of CD63 were found in the septic shock cohort, despite the limited number of patients analyzed for this marker.

HLA-DR downregulation in monocytes has been associated with myeloid cell dysfunction and correlated with the SOFA score [19, 20], as confirmed in our non-sepsis plus sepsis cohort (Fig. 1d, e and Table 1). To better understand the association between IL-1R2 expression and the functional state of monocytes in sepsis, we investigated the correlation between IL-1R2 and HLA-DR levels in monocytic cells. HLA-DR was negatively correlated with IL-1R2 (Spearman *r* = −0.48, *p* < 0.0001) (Table 1). In parallel, we observed a negative correlation of MS4A4A, used here as marker of differentiation, with HLA-DR (Spearman *r* = −0.26, *p* = 0.004) (Table 1). A weak but significant correlation of IL-1R2 with MS4A4A was found by evaluating all individuals enrolled in the study (Spearman *r* = 0.22, *p* = 0.015), whereas a strong correlation was observed in healthy donors and non-sepsis patients (Spearman *r* = 0.61, *p* < 0.0001), suggesting rapid and concomitant induction of the two molecules upon infection.

Collectively, these results suggest that the upregulation of IL-1R2 was associated with the acquisition of monocyte-to-macrophage differentiation markers (e.g., MS4A4A) and downregulation of HLA-DR.

### Regulation of IL-1R2 by CSFs and lipopolysaccharide

The differentiation of monocytes to macrophages is promoted by M-CSF and GM-CSF [28, 29], and increased levels of circulating CSFs were reported in sepsis [30].

To experimentally mimic the phenotypic transition from monocyte to macrophage, we investigated the phenotype of monocytes stimulated with M-CSF or GM-CSF for a time shorter than usual, e.g., 4 instead of 7 days, and in the presence of lipopolysaccharide (LPS). As shown in Fig. 2f, h, stimulation of monocytes with M- or GM-CSF was associated with the induction of IL-1R2, CD63, and MS4A4A. M-CSF stimulated cells expressed significantly higher levels of these proteins than GM-CSF stimulated cells (Fig. 2f–h). CD14^+^ HLA-DR^+^ cells were predominant after M-CSF stimulation (Fig. 2i bottom panels, 2j), and no significant changes in cluster frequency were observed after stimulation with LPS (Fig. 2j). In contrast, GM-CSF monocytic cells resulted in three distinct Mono/M*φ* clusters, namely HLA-DR^+^CD14^low^CD16^−^ (or HLA-DR^+^, in green), HLA-DR^-^CD14^+^CD16^−^ (or CD14^+^CD16^−^, in blue), and HLA-DR^-^CD14^+^CD16^+^ (or CD14^+^CD16^+^, in red) (Fig. 2i upper panels, Fig. 2k). The stimulation with LPS promoted the reduction of HLA-DR^+^ cell frequency (Fig. 2k), as observed in septic patients.

In addition, in line with previous studies [31], the activation of M-CSF-generated macrophages with LPS was associated with downregulation of IL-1R2 (Fig. 2l). In contrast, the stimulation with LPS of GM-CSF-generated macrophages triggered the upregulation of IL-1R2 (Fig. 2m). As reported in sepsis, IL-1R2 expression in HLA-DR^+^ monocytic cells was lower upon stimulation with LPS (Fig. 2m). These results indicated that LPS has a divergent effect on IL-1R2 in GM-CSF- and M-CSF-induced Mono/M*φ*. Combination of GM-CSF and LPS recapitulated the reduction of HLA-DR, the upregulation of IL-1R2, and other markers of monocyte-to-macrophage transition as observed in patients with sepsis.

To investigate the molecular mechanisms involved in IL-1R2 regulation, we took advantage of publicly available ChIP-seq data of monocytes and macrophages, finding a specific enrichment of the myeloid-specific transcription factors CEBPB and SPI1 on *IL1R2* in macrophages (Fig. S3). Moreover, recruitment of POLII and reduction of the inhibitory epigenetic modification H3K4me27 were observed (Fig. S3). Of note, increased accessibility of CEBPB and SPI1 binding sites was reported in monocytes from patients with sepsis [13]. These results suggest that CEBPB and SPI1 are involved in the regulation of *IL1R2* expression in macrophages, as well as in monocytes from patients with sepsis.

**Fig. 3:**
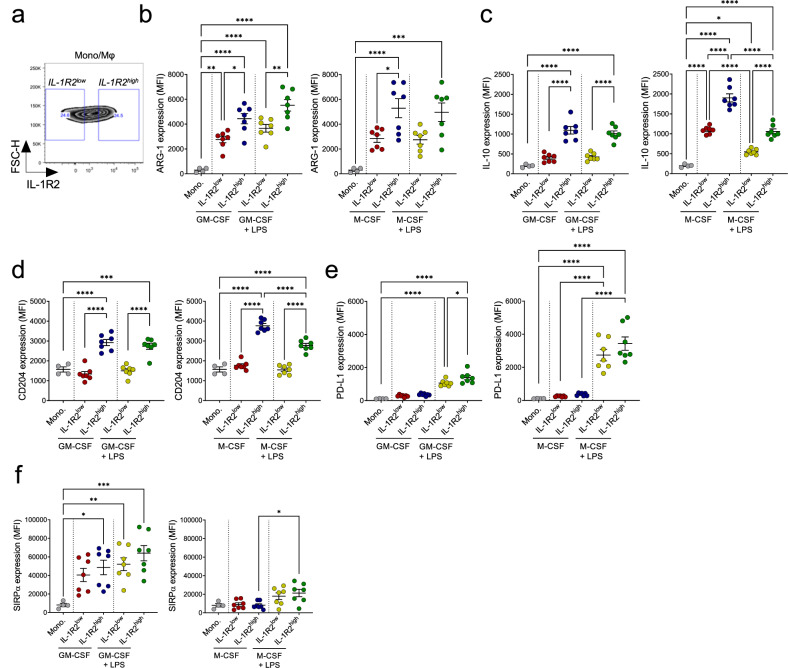
Markers of human IL-1R2^high^ Mono/M*φ* cells and clinical significance. **a** Gating strategy to identify IL-1R2^high^ and IL-1R2^low^ Mono/M*φ*. Expression of ARG-1 (**b**), IL-10 (**c**), CD204 (**d**), PD-L1 (**e**), and SIRPα (**f**) in monocytes, not polarized IL-1R2^high^ and IL-1R2^low^ Mono/M*φ* and Mono/M*φ* stimulated with LPS. GM-CSF Mono/M*φ* in the left panels; M-CSF in the right panels. **b**–**f** Data are shown as mean ± SEM. **b**–**f** One-way ANOVA with Tukey multiple comparison test, ^*^p < 0.05, ^**^p < 0.01, ^***^p < 0.001, ^****^p < 0.0001. *n* = 4 with technical duplicates of Mono/M*φ* for 3 donors. Pooled data from two experiments are shown.

To understand the biological significance of IL-1R2, we investigated the association between IL-1R2 and other functional markers of polarization, such as arginase-1 (ARG-1), IL-10, CD204, PD-L1, and SIRPα. As shown in Fig. 3a, monocytic cells differently expressed IL-1R2, but we did not identify separate IL-1R2^+^ and IL-1R2^−^ clusters. We thus compared the first quartile (Q1) expressing low levels of IL-1R2 (IL-1R2^low^) and the fourth quartile (Q4) expressing high levels of IL-1R2 (IL-1R2^high^) (Fig. 3a). ARG-1 (Fig. 3b) and IL-10 (Fig. 3c) expression was higher in IL-1R2^high^ compared to IL-1R2^low^, indicating a positive association between these immune suppressive molecules and IL-1R2. In addition, the differentiation marker and scavenger receptor CD204 (Fig. 3d) and the immune checkpoint (IC) PD-L1 (Fig. 3e) were upregulated in the IL-1R2^high^ subset. No differences were found between IL-1R2^high^ and IL-1R2^low^ on SIRPα, an IC specifically induced by GM-CSF but not by M-CSF (Fig. 3f). Interestingly, SIRPα was included among the scRNA-sequencing signature genes (FDR = 0.01) of sepsis monocytes [13].

Collectively, these results indicate that IL-1R2 is part of a signature associated with Mono/M*φ* differentiation (e.g., MS4A4A upregulation) preferentially in response to CSFs and LPS, including immunosuppressive markers, such as ARG-1, IL-10, PD-L1, and CD204.

### Membrane-associated IL-1R2 as a marker of sepsis

**Fig. 4:**
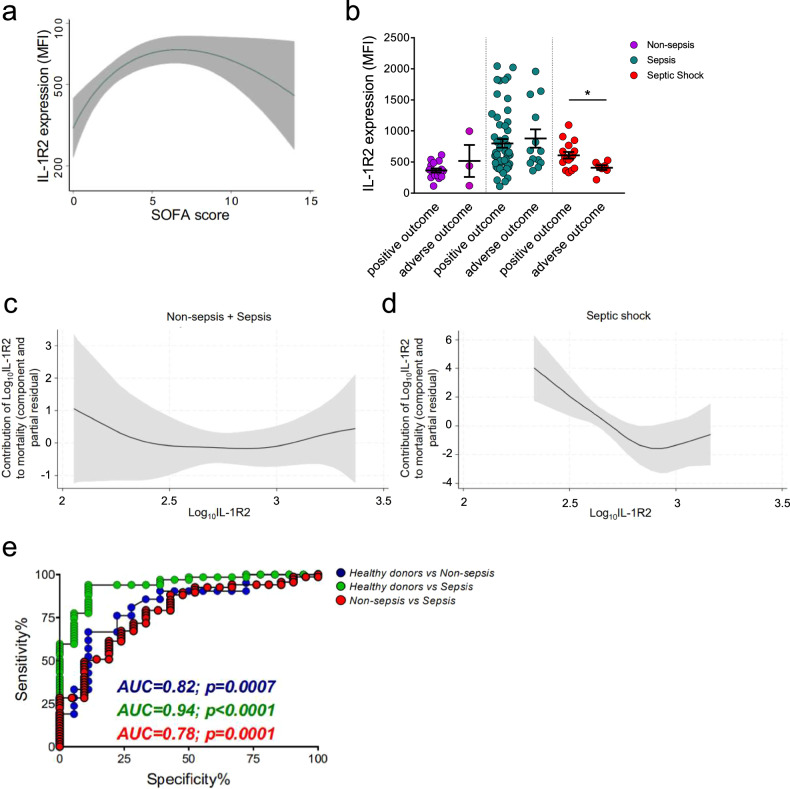
Association of monocyte membrane-IL-1R2 with mortality. **a** Association between the SOFA score and IL-1R2 expression on monocytes in the entire cohort. The gray area represents the 95% confidence interval. **b** Expression of IL-1R2 in monocytes from non-sepsis, sepsis, and septic shock patients with positive and adverse outcomes. Association of IL-1R2 in monocytes with survival in non-sepsis + sepsis (**c**) and septic shock (**d**) cohorts by using proportional hazards additive models with three degrees of freedom. The *y*-axis showed the partial contribution of IL-1R2 as a survival predictor. A value of 0 on the *y*-axis indicated no contribution to the model’s outcome. Positive values indicated that the predictor was associated with a higher outcome (mortality) within the cohort. **e** ROC curve analysis of IL-1R2. Blue line: ROC curve of healthy donors and non-sepsis patients; green line: ROC curve of healthy donors and septic patients; red line: ROC curve of non-sepsis and septic patients. **b** Two-tailed Mann–Whitney *U*-test. ^*^p < 0.05. **a**–**e** Healthy donors: *n* = 19; Non-sepsis patients: *n* = 23; Sepsis patients: *n* = 69; Septic shock patients: *n* = 25.

We next assessed the clinical significance of membrane-IL-1R2 in PBMCs. Progressively increasing monocyte IL-1R2 expression was observed in patients with SOFA score < 7 (Fig. 4a), reaching a plateau at this level of severity. High heterogeneity was observed in patients with SOFA score > 10. Monocyte IL-1R2 correlated with SOFA score especially in non-sepsis and sepsis cohorts (Spearman *r* = 0.47, *p* < 0.0001), faring more pronounced than HLA-DR as a biomarker of severity (Table 1). Statistically significant positive correlation was also observed in T and B lymphocytes from non-sepsis and sepsis cohorts (Table S3). No major differences were found stratifying patients by the site of infection origin and antibiotic treatment, as well as corticosteroid treatment, which is known to regulate IL-1R2 [32, 33] (Fig. S4). In addition, monocyte IL-1R2 significantly correlated with urea and creatinine (Table 1).

We next examined the association of IL-1R2 with pro-inflammatory cytokines and soluble mediators belonging to sepsis-associated cytokine storm [2, 12, 34]. The soluble and membrane-associated IL-1R2 were concomitantly upregulated (Fig. S5a), and positively correlated in non-sepsis and septic individuals (Table S4). Membrane-IL-1R2 expression was lower in monocytes of septic shock patients compared to sepsis, whereas sIL-1R2 plasma levels were higher [12], suggesting release from the cell membrane. However, we did not observe a negative correlation between the two forms in this cohort (Spearman *r* = 0.05, *p* = 0.63), suggesting that sIL-1R2 may be released from other cell types in addition to monocytes. IL-1R2 positively correlated with IL-6, IL-10, IL-8, and PTX3 [12] in non-sepsis and sepsis patients (Fig. S5b–i and Table S4).

To better elucidate the regulation of membrane-IL-1R2 in PBMCs in sepsis, we analyzed its expression by qPCR. *MS4A4A* and *CD14* were included as monocyte/macrophage reference genes. We observed a trend of increase in *IL1R2* mRNA levels depending on the severity, including in septic shock (Fig. S6a). The same trend was observed for *MS4A4A* (increase) and *CD14* (decrease) according to severity (Fig. S6b, c), and in agreement with flow cytometry results (Fig. 1). Collectively, these results suggest that post-transcriptional regulation and shedding of IL-1R2 are responsible for the downregulation of membrane-IL-1R2 expression and the increase of sIL-1R2 plasma levels.

Since MS4A4A was upregulated in patients with sepsis and correlated with IL-1R2 and HLA-DR, we evaluated its clinical significance. MS4A4A expression increased with the SOFA score as well (Fig. S7a), reaching its plateau at SOFA score 10. As observed for HLA-DR (Fig. 1e, f) and IL-1R2 (Fig. 4a), patients with SOFA score > 10 showed heterogeneous expression of MS4A4A (Fig. S7a). In agreement, MS4A4A positively correlated with the SOFA score in non-sepsis and sepsis subgroups (Spearman *r* = 0.3, *p* = 0.004), but not in the entire cohort (Table 1). Moreover, MS4A4A positively correlated with pro-inflammatory cytokines and sepsis-associated factors in the entire cohort (Table S4).

The clinical significance of IL-1R2 expression was investigated by stratifying patients according to outcome within 90 days of hospitalization. Membrane-IL-1R2 showed a significant reduction in septic shock patients with adverse outcome (Fig. 4b). Based on these results, we specifically investigated the correlation between IL-1R2, pro-inflammatory cytokines, and soluble factors in septic shock. Membrane-associated IL-1R2, but not sIL-1R2, negatively correlated with the IL-1 family members IL-1β and IL-18 (Table S5).

Septic shock patients with adverse outcome showed high levels of PTX3, among the inflammatory markers tested, in line with our previous study on the entire cohort [12] (Fig. S7b). Having found a correlation between membrane-IL-1R2 and soluble factors, Cox proportional hazards models were used after adjusting for IL-1-related biomarkers (i.e., sIL-1R2, IL-1β, IL-18, IL-1ra) and PTX3. IL-1R2 was not associated with survival in either the non-sepsis or sepsis cohorts (HR = 0.84, 95% CI: 0.10–6.77, *p* = 0.87). In contrast, a strong inverse association with survival emerged in patients with septic shock (HR = 0.02, 95% CI: 0.00–1.01, *p* = 0.05). These findings were further supported by proportional hazards additive models, which revealed a non-linear contribution of IL-1R2 to mortality in septic shock, but not in non-sepsis and sepsis cohorts (Fig. 4c, d).

Finally, we evaluated whether monocyte IL-1R2 expression stratified patients through a ROC curve analysis. IL-1R2 emerged as an accurate marker of disease discriminating healthy donors vs non-sepsis and sepsis subgroups, and stratifying non-sepsis and sepsis patients admitted to the emergency department (Fig. 4e). By comparison, the ROC curve analysis of MS4A4A expression showed that this marker discriminated healthy donors vs non-sepsis or sepsis groups (*p* < 0.0001), but did not stratify non-sepsis and sepsis patients (Fig. S7c).

Collectively, these results indicate that membrane-IL-1R2 on monocytic cells associates with markers of macrophage differentiation and dysfunction in sepsis. IL-1R2 expression positively correlates with the SOFA score and discriminates non-sepsis from sepsis individuals, whereas in septic shock, IL-1R2 is downregulated due to post-translational regulation, and it serves as a prognostic marker.

## Discussion

In this study, we show that IL-1R2 is upregulated in PBMCs of sepsis patients, in particular in monocytes characterized by low levels of HLA-DR, expression of macrophage markers, and sepsis-associated signature molecules, correlating with infection severity and cytokine storm. The IL-1R2^+^ monocyte phenotype was inducible in vitro upon stimulation of monocytes with CSFs, and in particular GM-CSF, in association with LPS.

We and others previously showed that circulating levels sIL-1R2 are increased in septic conditions, correlating with severity and mortality [12, 35–38]. Our analysis shows that membrane-IL-1R2 progressively increased from healthy donors to non-sepsis and sepsis patients, both in lymphocytes and monocytes. These findings are compatible with IL-1R2 transcriptional upregulation in sepsis [8]. We further observed a strong decrease of membrane-IL-1R2 in patients with septic shock, depending on post-transcriptional mechanisms. In agreement, the analysis of sIL-1R2 in plasma samples from the same cohort of patients showed progressively higher concentration correlating with sepsis severity, and persistence 5 days after admission [12].

Among PBMCs, the highest levels of IL-1R2 expression were observed in monocytes. Human monocytes were shown to downregulate IL-1R2 expression, both in persistent and resolving inflammation models [39]. Therefore, monocyte activation and polarization do not explain the increased expression of IL-1R2 in sepsis. Results presented here indicate that the molecular identity of monocytic cells was affected in sepsis and was associated with the acquisition of macrophage markers. Indeed, the sepsis monocyte MS1 signature [13, 40], which includes IL-1R2, was associated with the differentiation of circulating monocytes to macrophages. In line with this hypothesis, we showed that CEBPB and SPI1, reported as key transcription factors in monocytes from sepsis patients [13], interacted with *IL1R2* in macrophages and were associated with the POLII recruitment. In agreement with our results, IL-1R2 was identified as a Mono/M*φ* marker in bronchoalveolar lavage fluid (BALF) of COVID-19 patients [41], in partially-differentiated Dendritic Cells (DCs) generated in vitro [42], and in macrophages [43, 44].

CSFs play a key role in macrophage differentiation [45], and increased plasma levels have been found in infected individuals and septic patients [40, 46–48]. In addition, treatment with GM-CSF has been investigated in septic patients, with the aim of reversing sepsis-associated immune paralysis. On the other hand, studies targeting GM-CSF in COVID-19 are ongoing, with the aim to prevent immune hyperstimulation [49, 50]. Identification of a Mono/M*φ* signature in circulating cells opens a new perspective to interpret the effects of administration of CSFs or their blockade in severely infected patients.

In the sepsis cohort investigated in this study, we observed the recognized immunophenotypic features associated with sepsis, including lymphopenia and monocytosis, in combination with downregulation of HLA-DR [5, 51–53]. Downregulation of HLA-DR on monocytic cells correlated with severity, in contrast with other immunological parameters (e.g., CD4/CD8 lymphocyte ratio and monocyte/lymphocyte ratio). Alterations of monocytic cells from septic patients, which include IL-1R2 expression, have been associated with emergency myelopoiesis, highlighting the contribution of monocytes migrating from the bone marrow [13, 54]. Our results also reveal that circulating monocytic cells undergoing myeloid differentiation upregulate IL-1R2 and show a dysregulated immune phenotype associated with sepsis. We used CSFs to generate Mono/M*φ*, showing that the stimulation with GM-CSF plus LPS recapitulated, at least in part, sepsis-dependent alterations reported in patients. In particular, GM-CSF and LPS, but not M-CSF, induced strong downregulation of HLA-DR.

We associated IL-1R2^+^ HLA-DR^low^ cells generated in vitro with immune checkpoints and immunosuppressive molecules, in agreement with the MS1 signature in sepsis. Dissection of the immune phenotype in SARS-CoV2 infection revealed dysfunction of the myeloid cell compartment, associated with the presence of CD14^+^ HLA-DR^low^ myeloid-derived suppressor cells (MDSCs) in the peripheral blood of severe COVID-19 patients [41, 55], supporting the view that viral sepsis promotes the generation of immune suppressive cells [56]. IL-1R2 has been associated with functional impairment of myelomonocytic cells, including neutrophils [13, 14], but its direct role in the immune suppressive phenotype of myeloid cells in sepsis remains elusive.

IL-1R2 correlated with the SOFA score and HLA-DR, reflecting the severity of the condition. We further analyzed the significance of MS4A4A, a macrophage marker associated with IL-1R2 [21, 22]. MS4A4A was previously shown to be part of a set of genes upregulated in septic patients [27] and to promote Arg1 expression in mouse macrophages [57]. We found that MS4A4A negatively correlated with HLA-DR and positively with pro-inflammatory cytokines in septic patients, similarly to IL-1R2, in line with studies in mice [21]. IL-1R2 was shown to better stratify non-sepsis versus septic patients, while MS4A4A expression correlated with several cytokines and chemokines.

High heterogeneity in the expression of markers investigated in this study (IL-1R2, HLA-DR, MS4A4A, and cytokines) was observed in septic shock and critically ill patients with SOFA score > 10, leading to poor correlation when we included the septic shock subgroup in the statistical analysis. The heterogeneity of the most severe patients incited further statistical analysis to better associate them with the outcome. Using Cox proportional hazards models and proportional hazards additive models with three degrees of freedom, we found that membrane-IL-1R2, adjusted for clinically relevant plasma factors, served as a prognostic marker in patients with septic shock.

Thus, in sepsis, IL-1R2 is expressed in a subset of circulating monocytes co-expressing mature macrophage features with clinical significance and reflecting immune dysfunction.

## Supplementary information

Supplementary Figures and Tables

## Data Availability

The datasets analyzed during the current study is available from (https://www.proteinatlas.org/humanproteome/single+cell+type) (updated on 13/10/2022).
