# Supplementary material for: Monocyte-macrophage membrane expression of IL-1R2 is a severity biomarker in sepsis
Source: Cell Death Dis. 2025 Apr 10;16(1):269. doi: 10.1038/s41419-025-07597-x (PMC11982311; doi:10.1038/s41419-025-07597-x)
Supplement: Supplementary file 1 — Supplementary Figures and Tables [file 41419_2025_7597_MOESM1_ESM.pdf]

Monocyte-macrophage membrane expression of IL-1R2 is a severity biomarker in sepsis.

Domenico Supino* [1], Sadaf Davoudian* [1], Rita Silva-Gomes* [1,2,3], Daniele Piovani [1,4], Roberto Garuti [1,4], Antonio Desai [4,5], Sarah N Mapelli [1], Francesco Scavello [1], Silvia Carnevale [1], Andrea Mariancini [1,4], Elena Magrini [1], Roberto Leone [1], Marina Sironi [1], Sonia Valentino [1], Diletta Di Mitri [1,4], Federica Portale [1], Carlo Fedeli [5], Denise Comina [5], Stefanos Bonovas [1,4], Antonio Voza [4,5], Alberto Mantovani [1,4,6], Barbara Bottazzi [1], Cecilia Garlanda [1, 4 §]

[1]IRCCS Humanitas Research Hospital, Milan, Italy.

[2]Life and Health Sciences Research Institute (ICVS), School of Medicine, University of Minho, Braga, Portugal.

[3]ICVS/3B's-PT Government Associate Laboratory, Guimarães/Braga, Portugal.

[4]Department of Biomedical Sciences, Humanitas University, Milan, Italy.

[5]Department of Emergency, IRCCS Humanitas Research Hospital, Milan, Italy.

[6]The William Harvey Research Institute, Queen Mary University of London, London, United Kingdom.

* These authors contributed equally to this work

§ Correspondence: cecilia.garlanda@humanitasresearch.it

Associate Professor of Clinical Pathology

Department of Biomedical Sciences – Humanitas University

Head of Laboratory of Experimental Immunopathology – Humanitas Research Hospital

Humanitas University

Via Rita Levi Montalcini 20090 Pieve Emanuele (Milano) – Italy

+39 02 82245115

Supplementary Figures

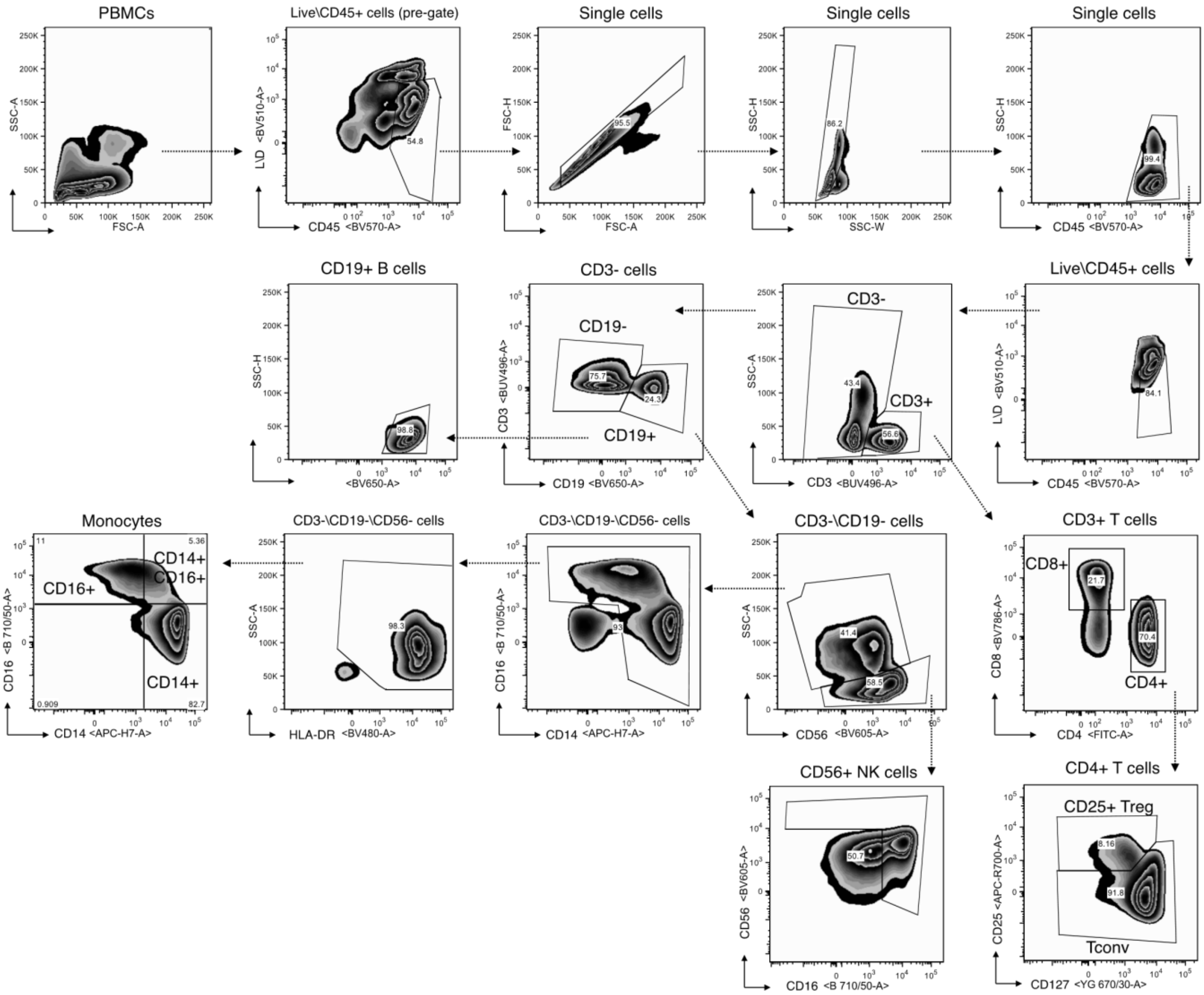

Figure S1. Gating strategy of human PBMCs.

Representative gating strategy for characterization of PBMCs.

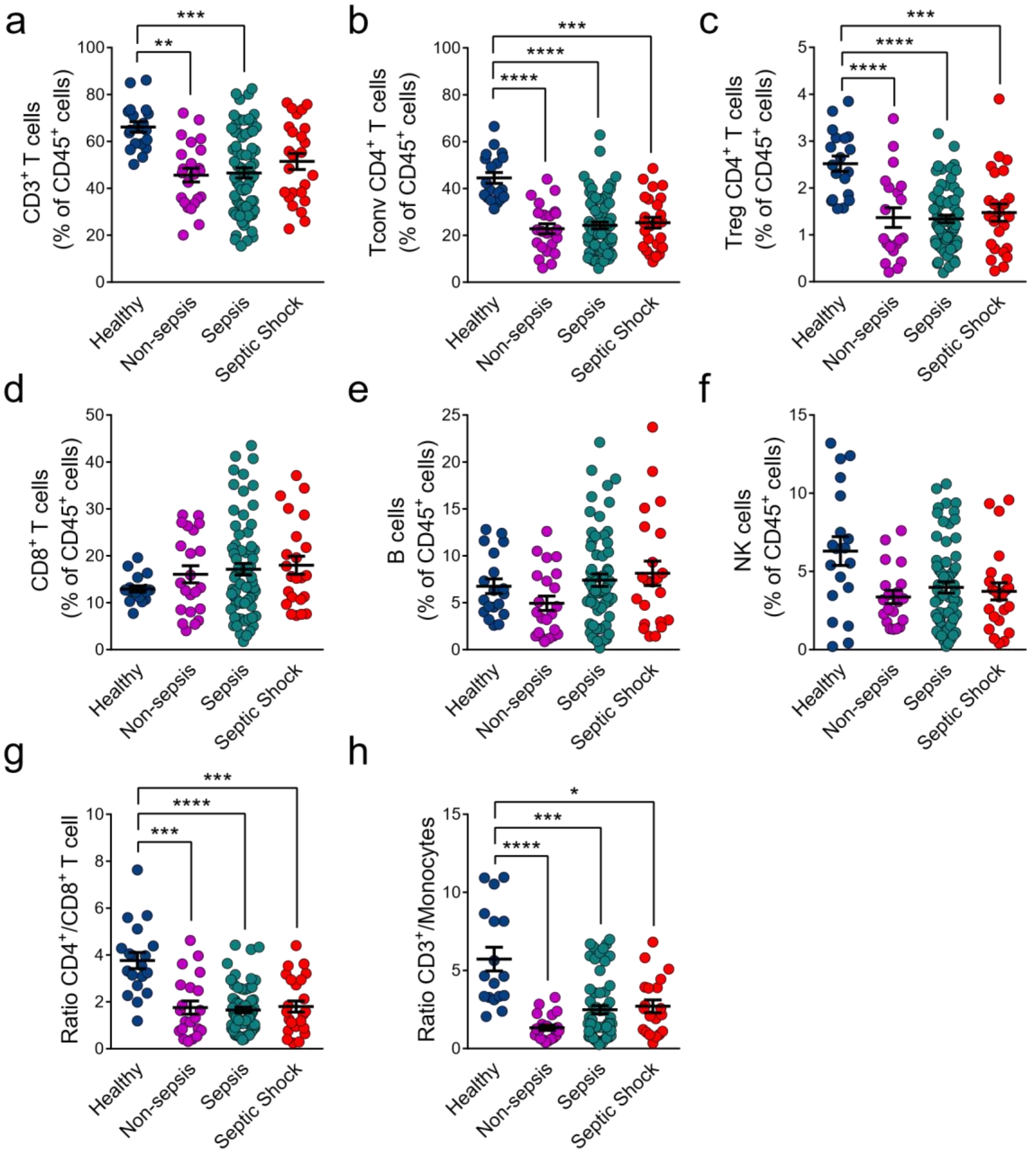

Figure S2. Immunophenotyping of lymphocytes from sepsis.

(a-f) Frequencies of CD3$^+$ T cells (a) and Tconv CD4$^+$ (b), Treg (c) and CD8$^+$ (d) T cell subsets, B (e) and NK (f) cells assessed by flow cytometric analysis. (g, h) Ratio of CD4$^+$ to CD8$^+$ T cells (g) and of CD3$^+$ cells to monocytes (h) in the entire cohort. One-way ANOVA with Tukey multiple comparison test (c) or Kruskal-Wallis with Dunn's multiple comparison test (a, b, d-h). **$p < 0.01$, ***$p < 0.001$, ****$p < 0.0001$. Healthy donors: n=19; Non-sepsis patients: n=23; Sepsis patients: n=69; Septic Shock patients: n=25.

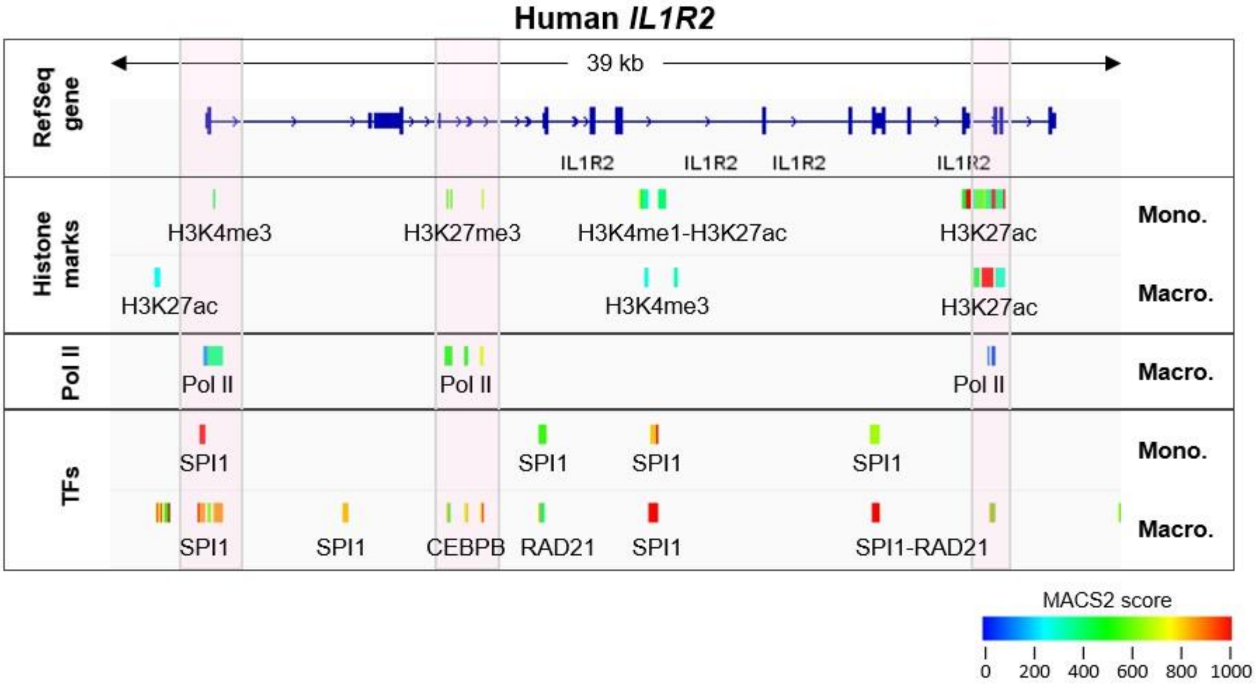

Figure S3. Epigenetic profiling of *IL1R2* in human monocytes and macrophages.

IGV plot showing histone marks (H3K4me1, H3K4me3, H3K27me3 and H3K27ac), RNA polymerase II (POL II) and selected Transcription Factor (TF) binding sites on *IL1R2* in human monocytes and macrophages. The genomic regions in which RNA polymerase II and TFs co-localized in macrophages are highlighted in red.

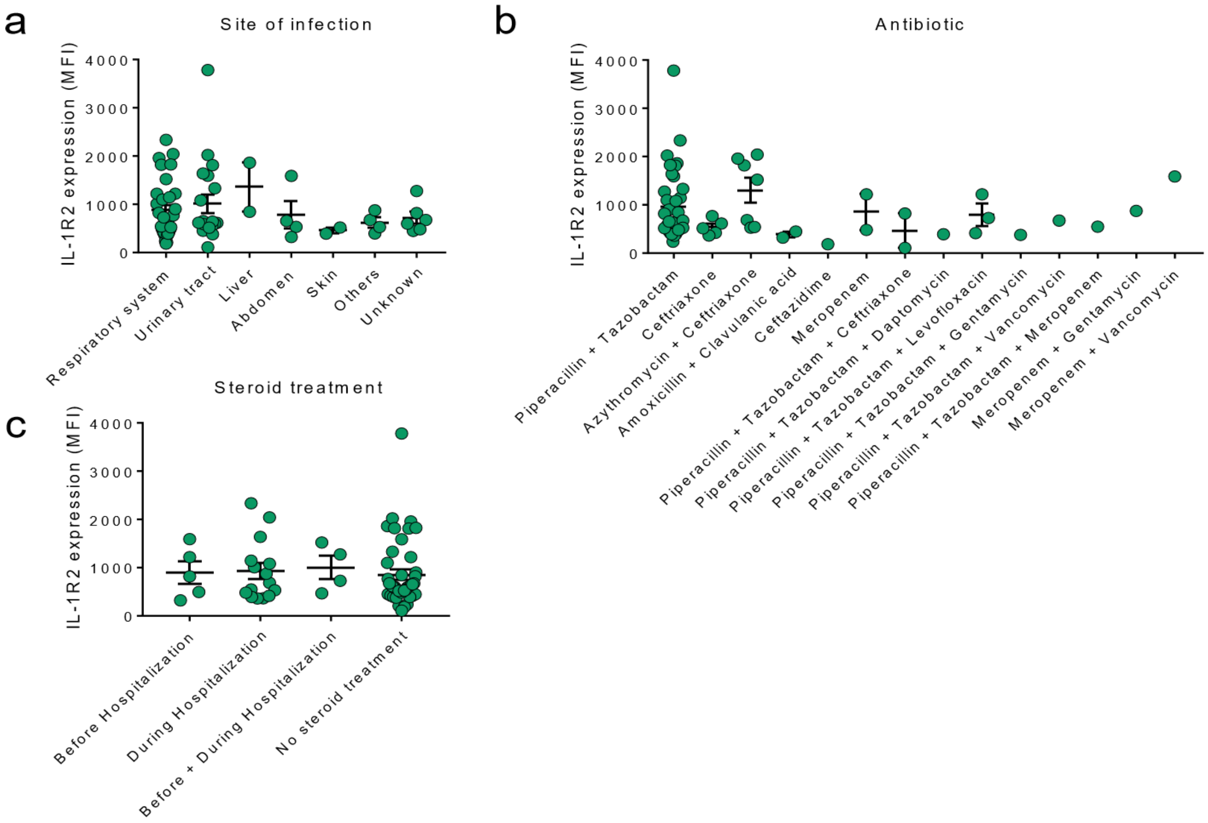

Figure S4. IL-1R2 expression and site of infection, antibiotic and steroid treatments.

(a-c) Expression of IL-1R2 on monocytes from sepsis patients stratified according to site of infection (a), antibiotic (b) and steroid (c) treatments. Kruskal-Wallis with Dunn’s multiple comparison.

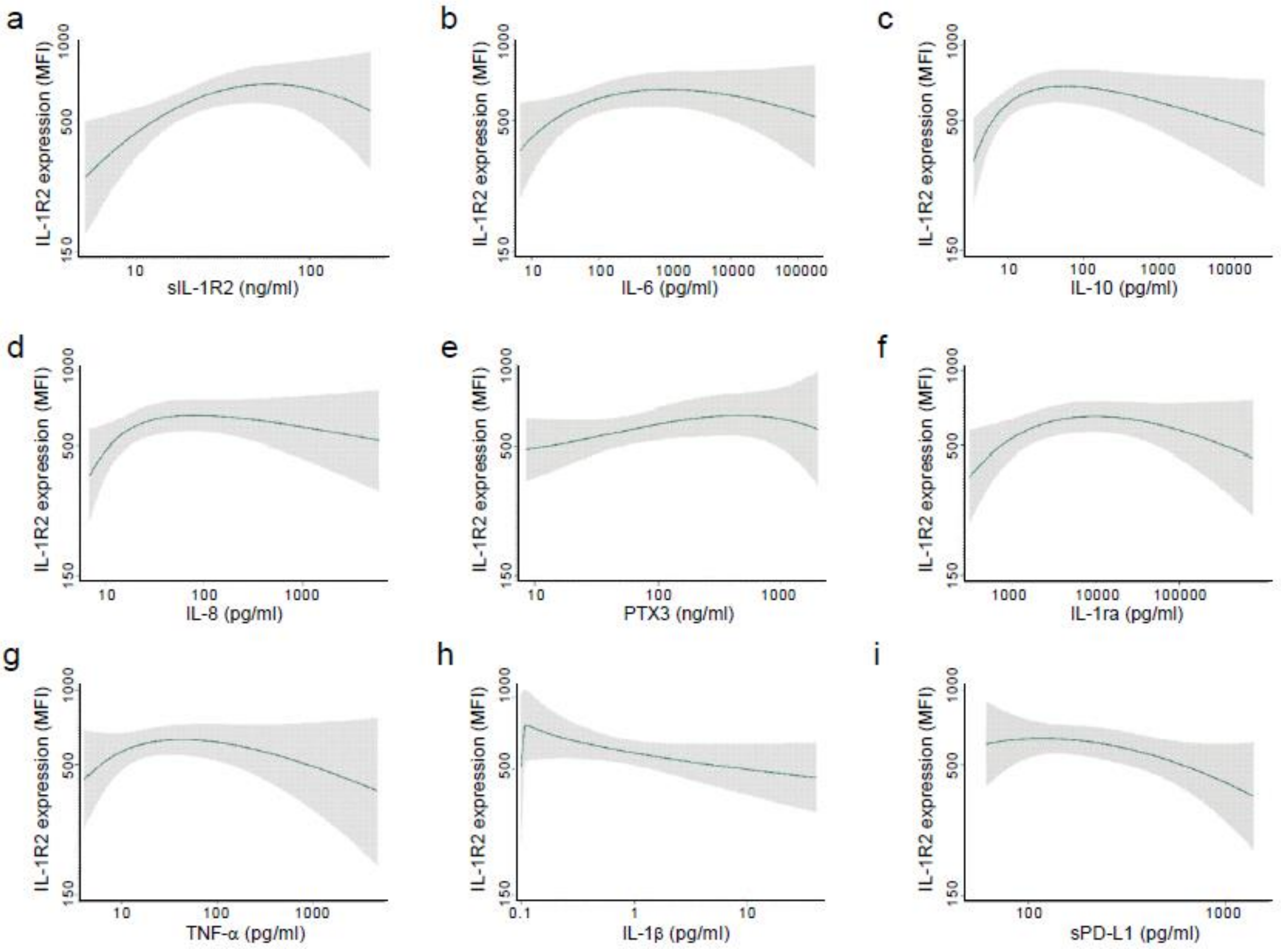

Figure S5. Association between IL-1R2 expression on monocytes, cytokines and immune factors.

(a-i) Association of soluble IL-1R2 (sIL-1R2) (a), IL-6 (b), IL-10 (c), IL-8 (d), PTX3 (e), IL-1 receptor antagonist (IL-1ra) (f), tumor necrosis factor α (TNF-α) (g), IL-1β (h) and soluble Programmed Death Ligand 1 (sPD-L1) (i) with IL-1R2 expression on monocytes (MFI) in the entire cohort. The gray area represents the 95% confidence interval.

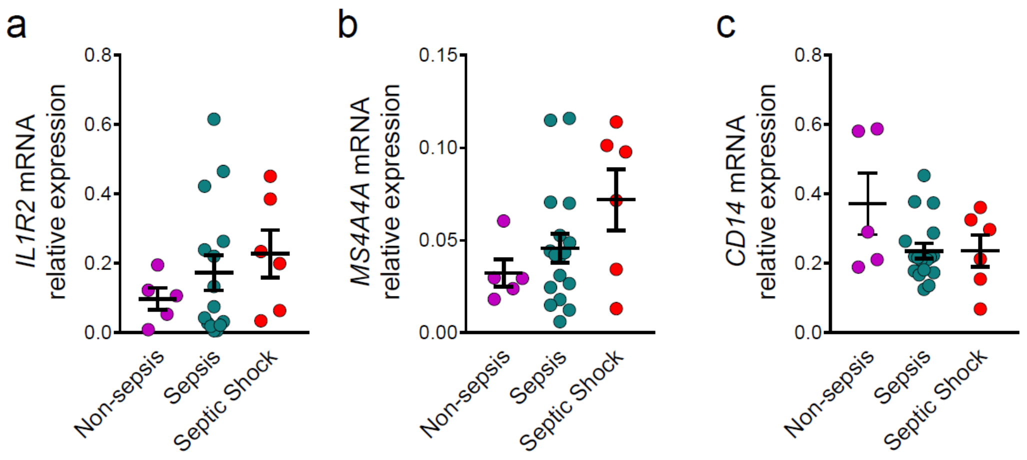

Figure S6. Characterization of *IL1R2, MS4A4A* and *CD14* gene expression profile in sepsis and septic shock patients.

(a-c) Expression of *IL1R2* (a), *MS4A4A* (b) and *CD14* (c) determined by qPCR. Gene expression was normalized on *GAPDH*. Non-sepsis patients (n=5); Sepsis patients (n=17); Septic shock patients (n=6). Kruskal-Wallis with Dunn's multiple comparison.

a

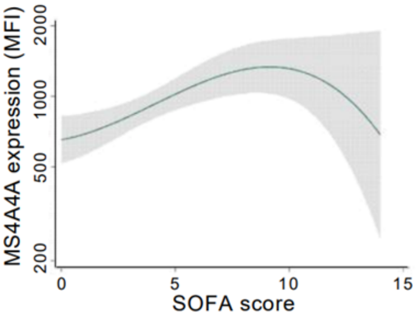

MS4A4A expression (MFI)
200
500
1000
2000
0
5
10
15
SOFA score

b

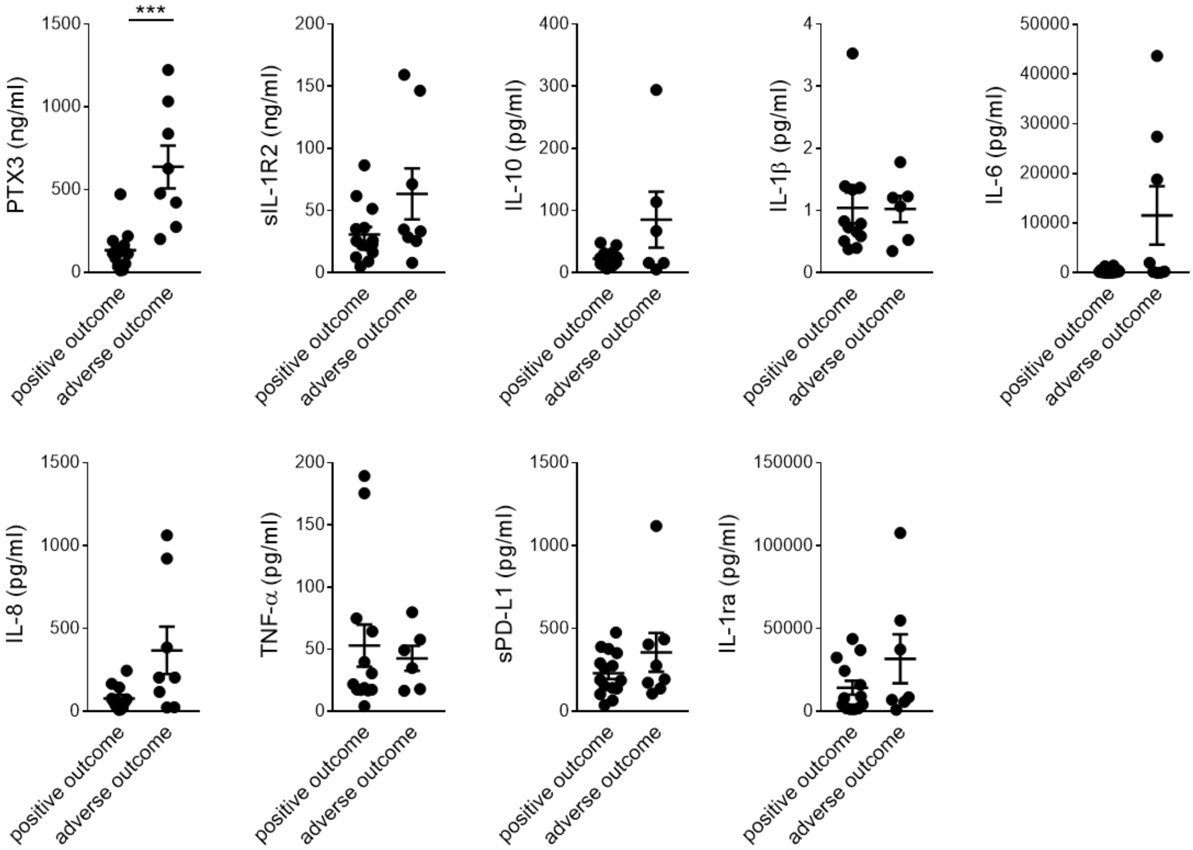

PTX3 (ng/ml)
***
sIL-1R2 (ng/ml)
IL-10 (pg/ml)
IL-1β (pg/ml)
IL-6 (pg/ml)
IL-8 (pg/ml)
TNF-α (pg/ml)
sPD-L1 (pg/ml)
IL-1ra (pg/ml)
positive outcome
adverse outcome

c

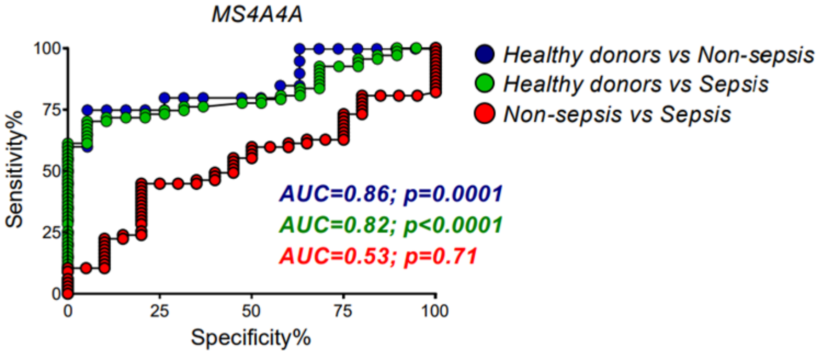

MS4A4A
Sensitivity%
Specificity%
Healthy donors vs Non-sepsis
Healthy donors vs Sepsis
Non-sepsis vs Sepsis
AUC=0.86; p=0.0001
AUC=0.82; p<0.0001
AUC=0.53; p=0.71

Figure S7. Clinical significance of MS4A4A and analysis of soluble factor levels in patients with positive and adverse outcome.

(a) Association between the SOFA score and MS4A4A expression on monocytes in the entire cohort. The gray area represents the 95% confidence interval. (b) Levels of PTX3, sIL-1R2, IL-10, IL-1β, IL-6, IL-8, TNF-α, sPD-L1 and IL-1ra in septic shock patients with positive and adverse outcome. (c) ROC curve analysis of MS4A4A. Blue line: ROC curve of healthy donors and non-sepsis patients; green line: ROC curve of healthy donors and septic patients; red line: ROC curve of non-sepsis and septic patients. (b) Two-tailed Mann–Whitney U-test. ***$p < 0.001$. (a-c) Healthy donors: n=19; Non-sepsis patients: n=23; Sepsis patients: n=69; Septic shock patients: n=25.

Supplementary Tables

Table S1 - List of flow cytometry antibodies used in this study.

Antibodies	Source	Cat #
Brilliant Violet 711™ anti-CD197 (CCR7) Clone G043H7	Bio legend	353228
Brilliant Violet 570™ anti-CD45 Clone HI30	Bio legend	304034
BV786 anti-CD8 Clone RPA-T8	BD	563823
FITC anti-CD4 Clone M-T477	BD	556615
BUV496 anti-CD3 Clone UCHT1	BD	564809
Brilliant Violet 605™ anti-CD56 (NCAM) Clone HCD56	Bio legend	318334
PerCP/Cyanine5.5 anti-CD16 Clone 3G8	Bio legend	302028
Brilliant Violet 421™ anti-CD279 (PD-1) Clone EH12.2H7	Bio legend	329920
PE anti-MS4A4A Clone 5C12/MS4A4A	Bio legend	372504
PE-Vio770 anti-CD121b (IL-1R2) Clone REA744	Miltenyi Biotec	130-111-273
PE-Cyanine5 anti-CD127 Clone eBioRDR5	eBioscience	15-1278-42
APCR700 anti-CD25 Clone 2A3	BD	565106
APC-H7 anti-CD14 Clone MφP9	BD	560180
BUV805 anti-CD14 Clone M5E2	BD	612902
Brilliant Violet 650™ anti-CD19 Clone HIB19	Biolegend	302238
BV480 anti-HLA-DR Clone G46-6 (RUO)	BD	566113
BUV661 anti-CD11b Clone M1/70	BD	565080\612977
Alexa Fluor™ 700 Arginase 1 Antibody Clone A1exF5	eBioscience	56-3697-80
BUV737 anti-CD172b Clone B4B6	BD	749203
BV421 anti-CD274 Clone MIH1	BD	563738
BV605 anti-MSR1 (CD204) Clone U23-56	BD	742440
BV650 anti-human and viral IL-10 Clone JES3-9D7	BD	564051
BV786 anti-CD63 Clone H5C6	BD	741004

Table S2. Demographic, laboratory and clinical characteristics of the total population stratified based on Sepsis-3 criteria.

Variable	Non-sepsis (n=21)	Sepsis (n=69)	Septic shock (n=25)	p-value*
**Age, median (Q1-Q3)**	72 [58-80]	77 [69-84]	78 [67-85]	0.16
**Gender, n (%)**				
**Male**	14 (67%)	45 (65%)	15 (60%)	
**Female**	7 (33%)	24 (35%)	10(40%)	
**Site of infection n (%)**				
**Respiratory system**	4 (19%)	31 (45%)	8 (32%)	**0.08**
**Urinary tract**	4 (19%)	20 (29%)	11 (44%)	0.17
**Liver**	2 (10%)	2 (3%)	1 (4%)	0.42
**Abdomen**	2 (10%)	4 (6%)	2 (8%)	0.47
**Skin and soft tissue**	1 (5%)	2 (3%)	1 (4%)	0.9
**Others**	4 (19%)	4 (6%)	0 (0%)	**0.04**
**Unknown**	4 (19%)	6 (9%)	2 (8%)	0.36
**Laboratory values, median [Q1-Q3]**				
**White blood cells (10^3/mm^3)**	11.06 [7.72-15.79]	11.63 [8.22-17.18]	15.21 [5.27-19.03]	0.81
**Lymphocytes (10^3/mm^3)**	0.7 [0.4-0.8]	0.65 [0.3-1.05]	0.6 [0.4-0.9]	0.86
**Neutrophils (10^3/mm^3)**	9.7 [6.3-14.4]	9.7 [6.9-15.65]	13.7 [4.9-15.8]	0.64
**Platelets (10^3/mm^3)**	200 [182-295]	170 [109-258]	163 [114-276]	0.11
**C-reactive protein (mg/dL)**	11.7 [4.36-19.56]	14.86 [6.63-24.11]	17.06 [12.26-21.93]	0.20
**Creatine (mg/dL)**	0.83 [0.55-0.96]	1.72 [1.03-2.45]	1.85 [1.24-3]	**0.0001**
**Hemoglobin (g/dl)**	11.4 [10-12.6]	11.7 [10.5-13.1]	11.6 [9.6-13]	0.64
**Hematocrit (%)**	35.4 [29.6-37.4]	35.4 [31.9-40]	35.5 [29.8-39.7]	0.61
**Sodium (mmol/L)**	137 [135-139]	136 [132-140]	137 [135-139]	0.43
**Potassium (mmol/L)**	3.8 [3.5-4.1]	3.9 [3.6-4.5]	4.2 [3.7-5.1]	**0.03**
**Urea (mg/dL)**	37.9 [30.7-47.1]	73.7 [47.15-118.2]	84.8 [59.8-126.4]	**0.0001**
**Fasting blood sugar (mg/dL)**	126 [109.5-156]	122 [104-167]	107 [90-193]	0.33

**Prothrombin Time (seconds)**	1.28 [1.16-1.48]	1.22 [1.14-1.45]	1.32 [1.17-1.95]	0.33
**Partial thromboplastin time (second)**	0.96 [0.9-1.04]	0.94 [0.87-1.03]	0.92 [0.84-1.1]	0.46
**Glasgow coma scale**	15 [15-15]	15 [15-15]	15 [13-15]	0.12
**SOFA score**	1 [1-1]	4 [3-6]	6 [4-10]	**0.0001**
**qSOFA score**	0 [0-0]	1 [0-1]	1 [1-2]	**0.0001**

**Kruskal-Wallis equality-of-populations rank test.*

Table S3. Correlation of IL-1R2 expressed by $CD3^+$ T cells and B cells with SOFA score.

Variables	Groups	Spearman r	p-value*
**IL-1R2 ($CD3^+$ T cells)**	Non-sepsis + Sepsis	0.4	**<0.0001**
	All infected patients	0.19	0.052
**IL-1R2 (B cells)**	Non-sepsis + Sepsis	0.37	**0.0003**
	All infected patients	0.16	0.1

**Spearman's rank correlation*

Table S4. Correlation analysis of IL-1R2, MS4A4A and HLA-DR on monocytic cells with soluble factors.

Variables	Groups	IL-1R2		MS4A4A		HLA-DR	
		Spearman r	p-value*	Spearman r	p-value*	Spearman r	p-value*
**sIL-1R2 (ng/ml)**	Non-sepsis + Sepsis	0.25	**0.017**	0.19	0.077	-0.3	**0.006**
	All infected patients	0.18	0.065	0.21	**0.027**	-0.23	**0.015**
**IL-10 (pg/ml)**	Non-sepsis + Sepsis	0.26	**0.016**	0.37	**0.0005**	-0.019	0.86
	All infected patients	0.18	0.057	0.33	**0.0004**	-0.012	0.9
**IL-1β (pg/ml)**	Non-sepsis + Sepsis	-0.017	0.87	0.42	**<0.0001**	0.047	0.67
	All infected patients	-0.14	0.15	0.36	**0.0001**	0.11	0.25
**IL-6 (pg/ml)**	Non-sepsis + Sepsis	0.28	**0.008**	0.43	**<0.0001**	-0.23	**0.031**
	All infected patients	0.16	0.097	0.4	**<0.0001**	-0.16	0.098
**TNF-α (pg/ml)**	Non-sepsis + Sepsis	0.14	0.18	0.54	**<0.0001**	0.077	0.48
	All infected patients	0.071	0.45	0.47	**<0.0001**	0.13	0.19
**sPD-L1 (pg/ml)**	Non-sepsis + Sepsis	-0.13	0.21	0.3	**0.0048**	0.19	0.085
	All infected patients	-0.084	0.38	0.29	**0.0022**	0.19	0.052
**IL-18 (pg/ml)**	Non-sepsis + Sepsis	-0.077	0.48	0.11	0.32	0.15	0.17
	All infected patients	-0.15	0.11	0.082	0.4	0.16	0.088
**IL-1Ra (pg/ml)**	Non-sepsis + Sepsis	0.21	0.051	0.37	**0.0004**	-0.034	0.76
	All infected patients	0.12	0.22	0.34	**0.0002**	0.03	0.74
**IL-8 (pg/ml)**	Non-sepsis + Sepsis	0.29	**0.0067**	0.39	**0.0002**	-0.15	0.18
	All infected patients	0.12	0.19	0.34	**0.0003**	-0.11	0.26
**PTX3 (ng/ml)**	Non-sepsis + Sepsis	0.22	**0.043**	0.35	**0.001**	-0.3	**0.0049**
	All infected patients	0.13	0.17	0.34	**0.0004**	-0.25	**0.0081**

**Spearman's rank correlation*

Table S5. Correlation of membrane-IL-1R2 and sIL-1R2 with soluble factors in septic shock patients.

Variables	Groups	Membrane-IL-1R2		sIL-1R2	
		Spearman r	p-value*	Spearman r	p-value*
**IL-10 (pg/ml)**	Septic Shock	-0.025	0.91	0.5	**0.012**
**IL-1β (pg/ml)**	Septic Shock	-0.62	**0.0013**	0.17	0.41
**IL-6 (pg/ml)**	Septic Shock	-0.23	0.28	0.46	**0.02**
**TNF-α (pg/ml)**	Septic Shock	-0.15	0.47	0.55	**0.0042**
**sPD-L1 (pg/ml)**	Septic Shock	0.15	0.49	0.53	**0.0067**
**IL-18 (pg/ml)**	Septic Shock	-0.42	**0.042**	0.63	**0.0007**
**IL-1Ra (pg/ml)**	Septic Shock	-0.16	0.46	0.53	**0.0064**
**IL-8 (pg/ml)**	Septic Shock	-0.4	0.056	0.57	**0.0027**
**PTX3 (ng/ml)**	Septic Shock	-0.14	0.51	0.42	**0.037**

**Spearman's rank correlation*
